# The Hospital of Medieval Venetian Methoni

**DOI:** 10.7759/cureus.43203

**Published:** 2023-08-09

**Authors:** Ioannis A Biris, Andreas I Biris, Spyros N Michaleas, Theodore G Papaioannou, Marianna Karamanou

**Affiliations:** 1 Department of History of Medicine and Medical Ethics, National and Kapodistrian University of Athens School of Medicine, Athens, GRC; 2 School of Medicine, University of Nicosia Medical School, Nicosia, CYP

**Keywords:** history of medicine, venetian senate, venetian colonies, venice, middle ages

## Abstract

In medieval Venetian Methoni, the provision of medical care could be roughly divided into two phases. During the first phase, Venice provided medical care solely to the staff, the garrison, and the Venetian citizens residing in Methoni. Medical care to the rest of the population was offered by the solitary orders that had settled in the area after the conquest of Methoni, in the context of charity. In the second phase, when trade with the East and also the pilgrimages to the Holy Places increased, the Senate took over medical care, initially by subsidizing the local monasteries and later, according to a decision made by the Senate of Venice since 1423, by taking over the provision from its own hospital, which was located in the residential area of the castle, dedicated to Saint Johannes the Theologian (Evangelist).

## Introduction and background

After the conquest of Constantinople by the Latin crusaders in 1204, Venice hurried to secure the sea routes of the eastern Mediterranean for its own interests. The geographical position of Koroni and Methoni was important for both the supply and repairs of ships and more generally for the safety of navigation in the eastern Mediterranean area. In 1209, with the Treaty of Sapienza, Venice kept the two Messinian ports under its control, ceding almost all of the rest of Morea to the French. Initially, the Senate’s interest was limited to the control of the two ports so that Venice could suppress the piracy that was flourishing in the eastern Mediterranean at the time [1].

Following the conquest of the port of Methoni by the Republic of Venice and the looming danger of Ottoman descent in the region, action had to be taken relating to the safety and social welfare of the passengers of the Venetian ships to the East. The safe operation of a hospital in Methoni was deemed necessary, and in 1423, the Senate ordered the castellano (commander) to transfer the hospital of Saint Johannes from the walled settlement outside the walls of the castle (burgo) to the residential area within the castle. The position of this hospital is highlighted here after a study of the sources as well as on-field research [1].

## Review

The hospital of Methoni during the Venetian era

After the conquest of the Messinian colonies and since the interest in travels and pilgrimage to the Holy Land began to grow, the Senate of Venice decided to organize its possessions there by strengthening its fortifications. From the beginning of the 12th century, with the blessings and encouragement of every pope, the visit to Jerusalem was a task and a life goal for many European Christian believers. Hundreds of people from France, England, Scotland, and Germany as well as Spain and the Low Countries flocked to set sail from the port of Venice for the journey to the East. Large galleys were sailing from the port of Venice for Jaffa, passing through the Greek seas. The itinerary was usual: Venice, Zara or Ragusa, Corfu, Methoni or Koroni, Crete, Rhodes, Jaffa. The passages between the northwestern coast of Crete and the Peloponnese as well as the one between eastern Crete and Rhodes, which were usually controlled by pirates, were of particular importance for the journeys to the Holy Land. Byzantium was also the crossroads for the trade routes of the East. The products from the Black Sea passed through the Bosporus and the Dardanelles, which were controlled by Constantinople, and through the Greek seas as they reached the West. The southern coasts of Crete were of similar commercial importance, as they controlled the route of spices in the passage from the Red Sea and Alexandria in Egypt to the markets of the West. The trips to the Holy Land had a mandatory stop at the two castle states of Messinia. Initially, after the conquest, there was a thought for the immediate demolition of the fortifications of the two Messinian cities, an idea that was rejected immediately. In the decade 1220-1230, the Serene Republic of Venice granted its possessions to tenants (affictatores) and later took them back under its direct control. In 1269, the Senate decided on the fortification of the ruined castle of Koroni and the additional construction of its fortification towers in 1283. In 1292, the construction of the fortress of Methoni also began [2].

After the completion of the fortifications, good administration (Statuto di Corone e Modone) turned the two Venetian possessions in Messinia into important commercial export stations that enjoyed great prosperity, after they became naval stations for the refueling and repair of passing ships. The colonization of the two cities became a matter of first priority for the metropolis. A condition for granting rights to the new inhabitants of the Messinian possessions was the award of Venetian citizenship (cittadinanza). Due to changes in the structure of Venetian society, Venice adjusted its policy by roughly dividing citizens into three categories. The first concerned citizens by blood (de iure sanguinis), i.e., the descendants of two generations of Venetian citizens who lived in the metropolis (cives originarii Venetiarum) or in the colonies (cives originarii terrarium et locorum nostrorum). Given the new administrative needs, two more categories of citizenship were created for foreigners in the colonies. Citizenship by grace (per gratiam) and that which concerned the inhabitants of the colonies to whom Venice granted protection (subditi). All types of citizenship were mainly intended to increase the population of the possessions and to also empower Westerners to settle in the colonies [3].

**Figure 1:**
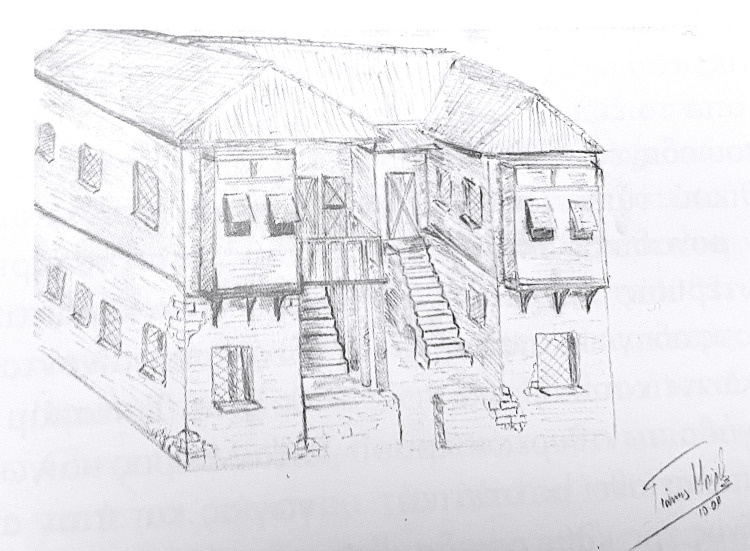
A virtual illustration of the medieval hospital of Venetian Methoni Source: Ioannis A. Biris. This is an open-source image reproduced under the Creative Commons license.

According to the model of the metropolis, as well as in the colonies of Venice, brotherhoods (Scuole) were operating in the parishes of the city’s temples. The three churches located within the walled settlement outside the castle of Methoni were dedicated to Saint Anna, to Saint Mary at the beach (Santa Maria alla spiaggia), and Saint Johannes. The locations of the two churches, that of Saint Anna and that of Saint Johannes, remain unknown to this day. The temple of Saint Mary existed up until the 19th century when it was demolished. According to a document of the Senate, the hospital outside the walls of the castle was dedicated to Saint Johannes [4] (Figure 1).

As per the aforementioned document, on the 6th of August 1423, Venice ordered the castellano of Methoni, Jacopo Erizzo, to transfer the hospital of Saint Johannes, from the settlement outside the walls of the castle to inside the castle. A rough translation follows:

*“The castellans of *Methoni*, present and future, along with two citizens will be administrators of the hospital of Saint Johannes of *Methoni*.*

Advisors.

*Despite a hospital already existing in our land *Methoni*, belonging to Saint Johannes, for the administration of which there are two loyal citizens who carry out their duties to the best of their abilities, nevertheless there are many inconsistencies, and it is best that in any case, the hospital must be managed properly and that the will of the founders and benefactors of the aforementioned hospital must materialize.*

*The part that belongs to the authority of this council will be passed on to the authority of our castellans in *Methoni​​​​​​​*, present and future.*

For those two loyal citizens, who will be appointed caretakers of the aforementioned hospital of Saint Johannes, the castellans will have to be administrators of this hospital and its goods, providing every care for the maintenance and the increase of wealth of this hospital and the poor people residing within it.

*As a result, in any case, without delay, it will be possible to carry out any deed that is in the glory of God’s name, the intention of the founders and benefactors of the hospital and to benefit the poor of this hospital, and that this site is added on the guardianship of our castellan in *Methoni​​​​​​​*, to maintain with all due respect” *[5].

The new hospital was then built in the residential area of the castle state, opposite the temple of Saint Johannes the Theologian, east of the main road, at the intersection that led to the southern quay of the port, next to the complex of towers of the Porta del Mandrachio. The layout, on an almost square plot of 38 by 36 meters, shows a “Π” - shape building for more sufficient ventilation of its spaces, with two parallel wings, with two also parallel ladders, in between them, which led to the hospital’s first floor [6,7].

**Figure 2:**
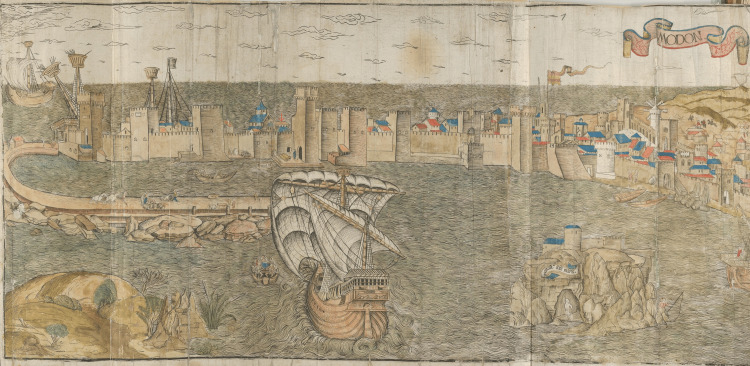
Erhard Reuwich, Modon. Woodcut published in 1486 in Bernhard von Breidenbach’s "Peregrinatio in Terram Sanctam" Source: Ioannis A. Biris, Methoni. Athens; 2005. This is an open-source image reproduced under the Creative Commons license.

**Figure 3:**
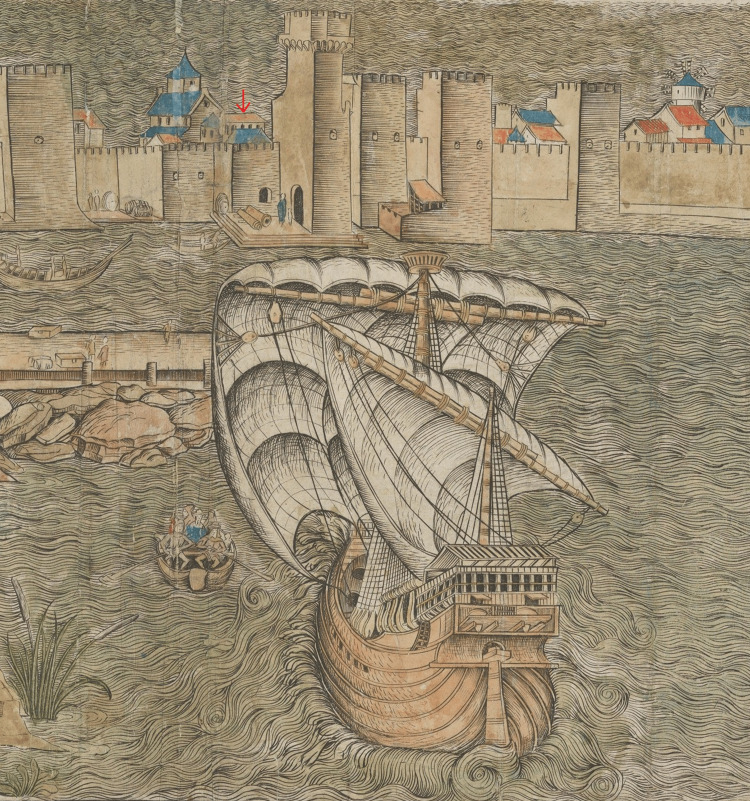
Detail of Reuwich’s, Modon. The roofs of the hospital can be seen between the church and the towers Source: Ioannis A. Biris, Methoni. Athens; 2005. This is an open-source image reproduced under the Creative Commons license.

In the first known depiction of Venetian Methoni (Modon) in 1486 in a woodcut by Erhard Rewich in Bernhard von Breydenbach’s “Peregrinatio in Terram Sanctam”, between the church and the complex of towers behind the walls, there can be found roofs of buildings that can be attributed as belonging to the hospital. The detailed illustration was made by Rewich on the 15th and 16th of June in 1483 [6] (Figures 2-3).

**Figure 4:**
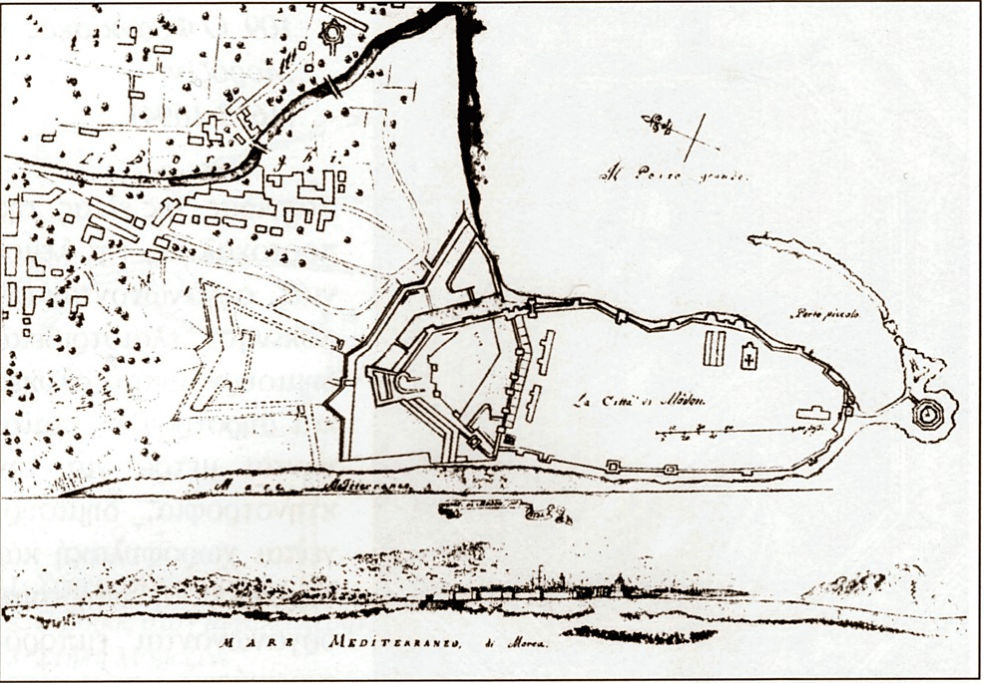
The location of the medieval hospital Source: Ioannis A. Biris, Methoni. Athens; 2005. This is an open-source image reproduced under the Creative Commons license.

The ruins of the hospital of Methoni can still be found today within the castle state. Due to the very dense construction of the settlement within the castle and the unsanitary conditions that prevailed there, the French expeditionary corps proceeded to demolish many buildings, among others, to prevent their habitation by the Greeks, following their departure [6]. For the development of the settlement of the Greeks outside the walls of the castle of Methoni, the Frenchman, then a lieutenant colonel, commander of the Engineers of the French expeditionary corps Joseph-Victor Audoÿ, on May 4, 1829, mapped out the small town of Methoni [8] (Figure 4).

The location of the medieval hospital is still evident today, in the ruins of the castle’s residential area. The arches that supported the ladders are also distinct. The hospital, with repairs and improvements operated for more than 400 years. The hospital’s position is also depicted in subsequent topographical plans of the second Venetian rule, especially in that of Grimani in 1731, with both the hospital and the church of Saint Johannes being clearly visible [9].

**Figure 5:**
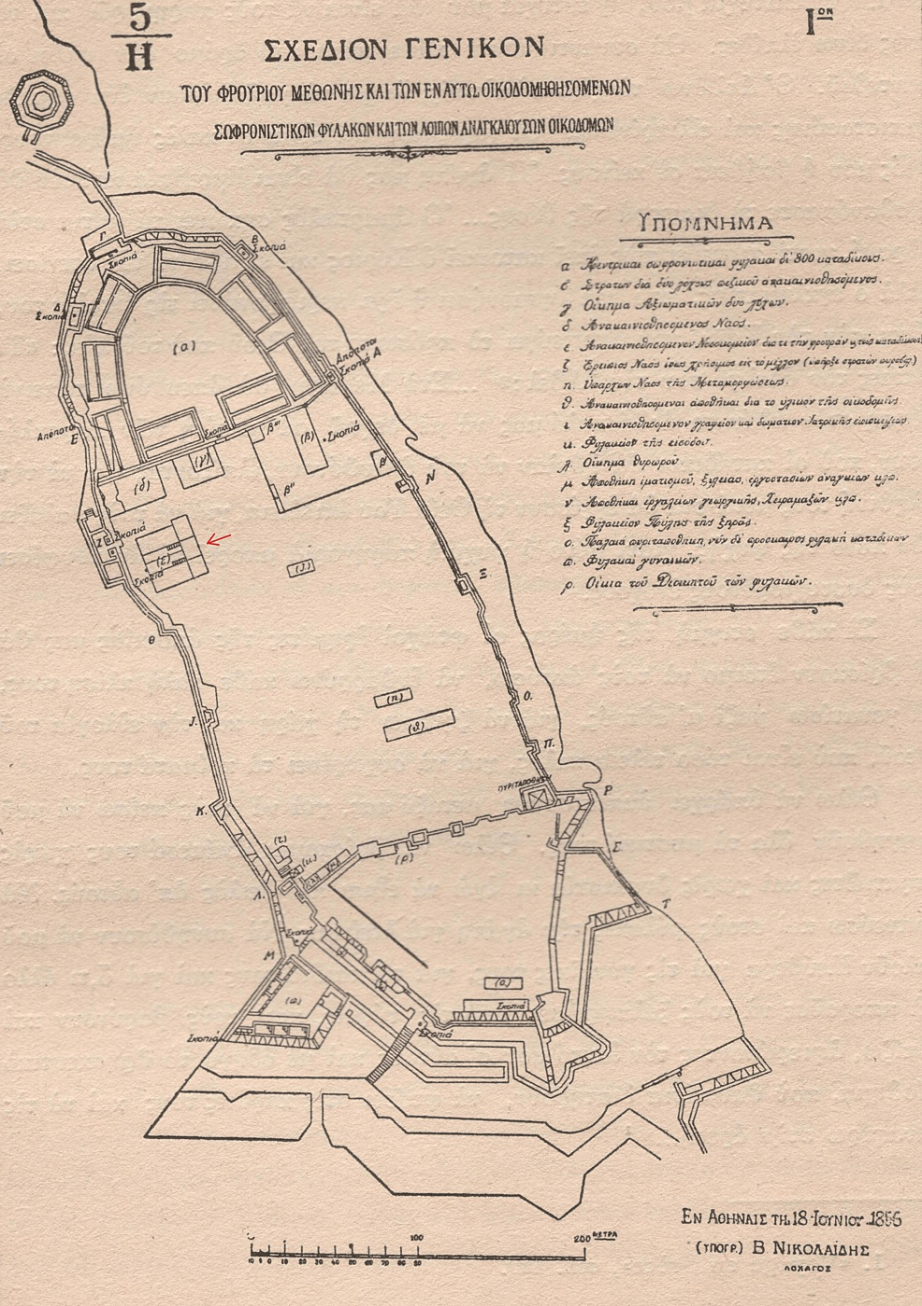
The location of the medieval hospital Source: Ioannis A. Biris, Methoni. Athens; 2005. This is an open-source image reproduced under the Creative Commons license.

The hospital is also mentioned in the proposal of the French knight Benjamin-Nicholas-Maria Appert to the King of Greece Otto I in 1856. Within this proposal, Appert proposed to the Greek government and the King, the creation of a penal colony within the castle of Methoni, with the application of mutual teaching amongst the convicts. In the memorandum of Appert’s plan, which accompanied his proposal, the sign “ε” has been put to illustrate the hospital’s position, along with the explanation “Renovated Hospital for the Guard and Convicts.” Appert’s proposal was, ultimately, not accepted [10] (Figure 5).

## Conclusions

The revelation of the medieval hospital of Venetian Methoni was based on the study of sources from the archives of Venice (Venetian archives) and the occasional depictions (illustrations) of the Venetian castle state. The decree of the Senate in 1423, Rewich’s woodcut in 1483, the site plans (topographical diagrams) of Grimani in 1731, and that of knight Appert in 1856 shed light on the ruins of the hospital of the medieval settlement within the castle of Methoni. It is possible that the excavation of the site could lead to the discovery of both the ruins of the hospital as well as medical equipment that was used at the time. Such findings would allow researchers to explore the literature and see how these tools were used at the time and how their use has evolved over the years.
